# Bilateral Seminoma in a Pediatric Patient With a History of Cryptorchidism: A Case Report

**DOI:** 10.7759/cureus.82080

**Published:** 2025-04-11

**Authors:** Erick A Rochel-Perez, Cesar J Ortiz-Castellanos, Ana S Hernandez-Martinez, Alejandro Martinez-Frias, Gustavo H Peniche-Gonzalez, Antonio Reyes-Cabrera

**Affiliations:** 1 Research, School of Medicine, Universidad Marista de Mérida, Mérida, MEX; 2 Research, School of Medicine and Health Sciences, Tecnologico de Monterrey, Monterrey, MEX; 3 Education, School of Medicine, Universidad Marista de Mérida, Mérida, MEX; 4 Pediatric Surgery, Hospital General Dr. Agustín O'Horán, Mérida, MEX; 5 Pathology, Hospital Regional ISSSTE (Instituto de Seguridad y Servicios Sociales de los Trabajadores del Estado), Mérida, MEX

**Keywords:** bilateral congenital cryptorchidism, bilateral orchiectomy, pediatric patient, testicular germ cell tumors, testicular seminoma

## Abstract

Synchronous bilateral testicular germ cell tumors (BTGCTs) are a rare subset of testicular germ cell tumors (GCTs) in pediatric patients. Cryptorchidism is a well-established risk factor for testicular cancer and markedly increases the risk of malignancy; thus, an early diagnosis is essential for effective management and prognosis. In this study, we discuss a case of a pediatric patient diagnosed with bilateral seminoma, treated surgically with bilateral orchiectomy. In this study, we describe a 16-year-old boy with a past medical history of bilateral cryptorchidism and orchidopexy, presenting with right testicular induration over the last month. Physical examination showed Tanner stage 3 genitalia with complete induration of the right testicle, but no palpable mass was detected in the left testicle. Scrotal ultrasound found heterogeneous nodules with increased vascularity, with microlithiasis involving bilateral testicles, prompting suspicion for a GCT. A bilateral orchiectomy with intraoperative biopsy was performed. Histopathological examination revealed bilateral seminoma, stage I, with the absence of any metastatic spread. Follow-up tumor markers and imaging study were unremarkable. In the pediatric population, bilateral seminoma is an extremely rare pathology with metachronous presentation more commonly described. The traditional risk factor of cryptorchidism remains important, highlighting the need for long-term follow-up of these patients. Diagnosis is based on clinical evaluation, imaging studies, and histopathological confirmation. Treatment usually consists of orchiectomy, with consequences for endocrine and fertility function. Due to the very low incidence of synchronous bilateral seminoma in this age group, a multidisciplinary approach is warranted with an emphasis on fertility preservation and hormonal follow-up. High-risk populations still require early detection via testicular ultrasound.

## Introduction

Germ cell tumors (GCTs) comprise a heterogeneous group of neoplasms with distinct histopathological subtypes, including seminoma, teratoma, choriocarcinoma, and yolk sac tumor. Testicular GCTs represent approximately 1% of all malignancies in males, with only about 7% of cases occurring in the pediatric population. Bilateral testicular GCTs are rare, accounting for only 0.5% to 1% of all testicular tumors. Approximately 80% of GCTs are benign, while the remaining 20% are classified as malignant [1,2]. The prognosis for patients with early-stage disease is favorable, with survival rates reaching up to 95%, primarily due to timely surgical resection of the affected testicle [2,3]. Although the etiology of GCTs remains largely unknown, several risk factors in pediatric patients have been associated with their development [3,4], including the following factors: (1) history of cryptorchidism: the risk of developing testicular cancer is 10 to 40 times higher in individuals with an undescended testicle. (2) Environmental exposure: exposure to organochlorines, biphenyls, polyvinyl chlorides, marijuana, and tobacco increases the risk of developing testicular cancer. (3) Infections: the antecedent of paramyxovirus infection increases the risk of developing testicular cancer. (4) Genetic factors: genetic mutations in BRAF, KIT, KRAS, NRAS, and TP53 genes have been identified as risk factors for developing testicular cancer.

Seminomas are a type of germ cell neoplasm with an estimated incidence of 0.9 cases per 100,000 children up to 15 years old. These tumors typically present clinically as a painless testicular mass with persistent swelling and, in rare cases, hydrocele or hematoma, usually affecting only one testicle. The use of biomarkers such as alpha-fetoprotein, beta-human chorionic gonadotropin, and lactic dehydrogenase would be useful in the diagnosis of germ cell testicular tumors. Nevertheless, pure seminomas do not elevate alpha-fetoprotein, the lactic dehydrogenase is nonspecific, and the beta-human chorionic gonadotropin is elevated occasionally in pure seminomas, making the diagnosis of this type of cancer more challenging [5,6].

In this study, we discuss a case of a pediatric patient diagnosed with bilateral seminoma, treated surgically with bilateral orchiectomy.

This case was presented as a poster at the XXI International Congress of Pediatrics, College of Pediatricians of Yucatán.

## Case presentation

A 16-year-old male patient presented to the pediatric clinic accompanied by his parents. During the anamnesis, he reported a one-month history of painless induration and mild swelling of the right testicle. He denied associated symptoms such as fever, weight loss, or systemic complaints. His relevant medical history included bilateral cryptorchidism, for which he underwent orchiopexy at one year of age. There was no known family history of GCTs.

On physical examination, the patient’s vital signs were within normal limits (Table 1), suggesting the absence of systemic involvement. Genital examination revealed Tanner stage III genitalia. There was induration and mild swelling of the right testicle, while the left testicle was unremarkable, with no palpable masses. No inguinal lymphadenopathy or other significant findings were noted.

**Table 1:** Vital signs of the patient recorded during the first consultation.

Vital signs		
Evaluation	Patient's value	Normal range
Height	159 cm (centile 3)	Centile 3 to centile 97
Weight and body mass index (BMI)	61 kg and 24.1 kg/m^2 ^(centile 85)	Centile 3 to centile 97
Cardiac rate	72	60-100 beats per minute
Blood pressure	110/73	Systolic pressure: 120 mmHg. Diastolic pressure: 80 mmHg
Respiratory rate	14	12-20 breaths per minute
Temperature	36.8ºC	36.1ºC-37.5ºC

A high-frequency linear transducer ultrasound (US) was performed on both testicles. The right testicle exhibited heterogeneous echogenicity with increased vascularity, three heterogeneous nodules with echogenic and hypoechoic solid areas, as well as predominant microlithiasis in the tail of the epididymis. The left testicle showed heterogeneous echogenicity with a hypoechoic nodule containing fine septations, areas of increased vascularity, and significant microlithiasis. The epididymis was dilated, with anechoic nodular formations in the head and body. The findings in both testicles were suggestive of a GCT; however, seminoma was not initially suspected.

Based on the ultrasound findings, the medical team decided to perform a bilateral orchiectomy with intraoperative biopsy of the left testicle. Prior to surgery, a complete blood count and coagulation profile were obtained, both yielding results within normal ranges, thus allowing the procedure to proceed without contraindications. An abdominal and pelvic computed tomography (CT) scan was also performed, revealing bilateral testicular tumors with calcifications, but no evidence of metastatic disease. A right orchiectomy was carried out with high ligation of the spermatic cord. This was followed by an intraoperative biopsy of the left testicle, and subsequently, a left orchiectomy with high ligation of the testicular vascular bundle (Table 2).

**Table 2:** Results of the pre-surgical tests performed on the patient.

Pre-surgical laboratory results		
Hematic biometry		
Evaluation	Patient's value	Normal range
Hemoglobin	16 g/dL	13.0-16.0 g/dL
Hematocrit	47%	38-49%
Total leukocytes	8,680 cells/mm³	4,500-11,000 cells/mm^3^
Neutrophils	66%	45-75%
Platelets	362,000 cells/mm³	150,000-450,000 cells/mm³
Coagulations test		
Prothrombin time	14.6 seconds	10-14 seconds
Partial thromboplastin time	32.5 seconds	25-35 seconds

Histopathological report

**Figure 1:**
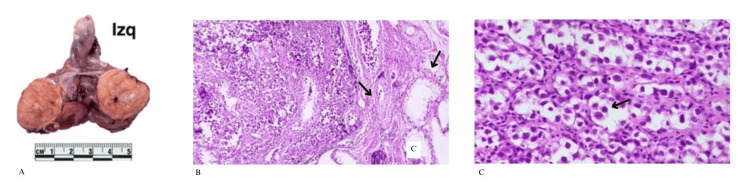
(A) The left testis measures 4.0 x 3.5 x 3.0 cm. On sectioning, testicular parenchyma is observed with a central fibrous area that appears white and of increased consistency. (B) The left testis shows areas of fibrosis with microcalcifications. At the periphery, seminiferous tubules are expanded by germ cell neoplasia in situ and the presence of a germ cell tumor of seminomatous type. (C) Large polygonal cells are observed with clear cytoplasm and prominent central nuclei, some with nucleoli and fine chromatin, along with interspersed lymphocytes.

The left testicle showed areas of fibrosis with microcalcifications and in situ neoplasia of seminomatous germ cells. The malignant cells were observed in sheets and nests of large cells with clear cytoplasm and prominent nuclei, surrounded by lymphocytes. No malignant cells were identified in the rete testis, epididymis, or spermatic cord (Figure 1).

**Figure 2:**
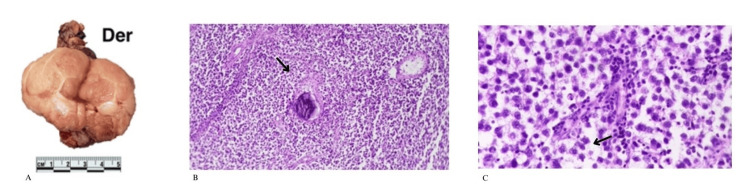
(A) The right testis is covered by a thin layer of connective tissue with no apparent lesions. On sectioning, a well-demarcated, lobulated neoplasm is identified, grayish-white in color, with an edematous appearance and thin septa. (B) The right testis shows infiltration by a neoplasm composed of sheets of polygonal cells with well-defined cytoplasmic borders. The neoplasm is divided into lobules by thin fibrous septa, with the presence of microcalcifications. (C) Polygonal cells are observed with well-defined cytoplasmic borders, clear cytoplasm, and round nuclei with central nucleoli.

The neoplasia in the right testicle had similar characteristics, consisting of polygonal cells with clear cytoplasm and well-defined borders. The tumor was lobulated and separated by fibrous septa with microcalcifications, extending to the rete testis and tunica albuginea without invasion of the tunica vaginalis. The epididymis and spermatic cord were free of lesions (Figure 2).

The histopathological findings confirmed a bilateral seminoma, with in situ neoplasia in the left testicle and invasive seminoma confined to the testis in the right. The absence of invasion into surrounding structures classifies the disease as stage I, associated with an excellent prognosis. These findings justified the bilateral orchiectomy, especially due to the germ cell neoplasia in situ, which carries a high risk of progression to invasive cancer. The patient will require lifelong testosterone replacement therapy and oncologic follow-up. A non-contrast abdominal and pelvic CT scan, alpha-fetoprotein (AFP) levels, and beta-human chorionic gonadotropin (β-hCG) levels were requested. All three tests yielded normal results, ruling out the possibility of metastasis to the lymphatic system or adjacent tissues.

## Discussion

In the United States, cancer is the second leading cause of death in children aged one to 14 years and fourth among adolescents aged 15 to 19 years. In 2024, the number of new cases was 9,620 and 5,290 in children and adolescents, respectively, with respective deaths of 1,040 and 550. One in about 257 under-20-year-olds will be diagnosed with cancer [7]. Mexico has reported yearly about 150 new cases of childhood cancer per million inhabitants [8], having experienced major alterations over the last 20 years in the incidence, prevalence, survival, and mortality rates of childhood cancer. These epidemiologic data highlight the importance for pediatricians to know the risk factors associated with GCT and their clinical presentation, given their presence in our setting and the clear benefits of early diagnosis and timely treatment.

Testicular tumors are unusual among adults, accounting for less than 1% of all cancer cases. They are mostly analogous to GCTs [9]. GCTs are rare in prepubertal patients, comprising only 1% of all pediatric solid tumors [10]. Bilateral testicular germ cell tumors (BTGCTs) account for 0.5% to 5% of all testicular tumors, with the majority being metachronous (a tumor occurring at a different time). In contrast, synchronous bilateral testicular germ cell tumors (SBTGCTs) occur in about 0.5% to 1% of cases [9,11]. Given this context, we highlight the unusual presentation of this case involving a synchronous bilateral testicular seminoma. Pediatric patients presenting with testicular induration or swelling, regardless of the absence of pain, should prompt consideration of this rare but significant diagnosis, as early detection is key to improving patient outcomes.

In this patient, there was cryptorchidism that was surgically corrected, which is a known risk factor for the development of testicular tumors (10-fold increased risk of malignancy over the general population) [12]. The association between cryptorchidism and GCTs has been described in several studies, with an estimated 5-10% of testicular cancer patients having suffered from cryptorchidism [13]. Testicular seminoma is commonly diagnosed using clinical examination and imaging, including scrotal ultrasound and serum tumor markers (AFP, β-hCG, and lactate dehydrogenase) [14]. In this case, testicular ultrasound proved to be a valuable tool in the diagnosis of GCT. The findings support the recommendation that ultrasound should be the initial imaging modality when there is a clinical suspicion of testicular tumors.

Bilateral orchiectomy and intraoperative biopsy confirmed histopathological diagnosis of stage I seminoma, without invasion of adjacent structures. In contrast, BTGCTs are to be managed differently since bilateral orchiectomy leads to hypogonadism and azoospermia, thus requiring endocrinological follow-up and consideration of fertility preservation options [15]. We could only detect a few cases of synchronous bilateral seminoma by reviewing the literature; therefore, our report illustrates how rare this clinical entity is and emphasizes the importance of multidisciplinary management and long-term follow-up.

## Conclusions

This case highlights the rare occurrence of synchronous bilateral testicular seminoma in a pediatric patient with a history of bilateral cryptorchidism, reinforcing the long-term malignancy risk associated with this condition despite early orchiopexy. The absence of systemic symptoms and normal tumor markers (AFP and β-hCG) posed a diagnostic challenge, emphasizing the critical role of testicular ultrasound in early tumor detection.

Definitive surgical management with bilateral orchiectomy revealed a stage I diagnosis, with no evidence of metastatic spread on imaging or biomarker elevation. This outcome underscores the importance of timely intervention and multidisciplinary management in optimizing prognosis. Given the complications of bilateral orchiectomy on hormonal and reproductive health, long-term endocrine follow-up and fertility counseling are essential considerations for comprehensive patient care.
